# Experience of video consultation during the COVID-19 pandemic in elderly population for Parkinson’s disease and movement disorders

**DOI:** 10.1136/postgradmedj-2020-138846

**Published:** 2020-10-02

**Authors:** Anil Kumar

**Affiliations:** Care of Elderly, University Hospitals of North Midlands NHS Trust Children’s Centre, Stoke-on-Trent, UK; Honorary Clinical Lecturer School of Medicine, Keele University, Keele, UK

**Keywords:** Geriatric medicine, Neurology, Parkinson’s disease

## Abstract

We have not been prepared for the current pandemic which has hit us hard. COVID-19, being a very contagious disease, one has to be very careful and diligent in caring for our patients keeping safety in mind all the time. For day-to-day care, new norms have been adopted for inpatient care. For outpatient care, the face-to-face (F2F) clinics were cancelled and instead telephone consultations were started. However, it has its own limitations. Unfortunately, there were patients who would need F2F consultation but could not come to the clinic due to the infection risks. For those patients, video consultation was started, which was an innovation in practice. The National Health Service has an Attend Anywhere clinic, as part of transformation in service, which enabled remote consultation with a better outcome than telephone clinics. However, it has its own limitation as not everyone could use it.

## INTRODUCTION

The current pandemic is unprecedented and unparalleled.

Since the middle of March 2020, face-to-face (F2F) consultations has been cancelled in view of the pandemic. However, alternative models of patient care, that is, telephone or video consultation has started. These alternative clinics are quite innovative and intuitive, and the success depends upon the maturity of the clinician doing the consultation. As one is not seeing the patients, the probing questions need to be very discriminative and targeted to establish or refute a diagnosis or clinical condition. Developing finesse and skill leading to a better outcome on physical examination is a new skill that is evolving. With ongoing pandemic, we are learning new ways of managing patients, and video consultation is one of them.

## STUDY

Since early April 2020, telephone clinics have been running smoothly, managing a reasonably good number of patients with the aforementioned skills. Elderly patients are quite comfortable to use this service too. In our study, we identified 11 patients who were not appropriate for telephone clinics as it needed a new diagnosis of Parkinson’s disease (including movement disorders) or initiation of medications; traditionally, these patients would have been seen in F2F clinics. If we waited for traditional outpatient departments (OPDs), this would lead to the danger of breaches in the referral to treatment. Besides this, we have a duty of care to look after patients in reasonably good time. In these circumstances, we offered to do video clinics instead. Of the 11 patients, 4 were willing to go for video consultation with the assistance of their family members, 1 was keen to do it himself and the remaining 6, however, preferred F2F consultation in the traditional OPDs.

As part of National Health Service (NHS) transformational change, training was imparted to the clinician for video consultation called ‘Attend Anywhere’ clinic (AA clinic). Patients were given written informations. Although not ideal, it was much better than telephone clinics.

### Advantages of video consultation

1. It allows the doctor and the patient to see each other.
2. It uses both verbal and non-verbal cues.
3. It uses both verbal and non-verbal information.
4. It allows some focused clinical examination.
5. It also helps with better visualisation of their mental health in the lockdown.

### Following were the concerns among the NHS staff in terms of AA clinic

1. Regarding our group of patients
  - Patients may not be able to handle the smartphone or the laptop.
  - Their dexterity with fingers may be problematic.
  - Their vision and hearing may be impaired.
  - The group is shielding with no family members visiting them, so may not get assistance.
2. It could be a logistical challenge as well as waste of training time, equipment and the efforts to organise this.

### Our experience

As this was a new use of AA clinic, we were a bit apprehensive but excited and enthusiastic as we were trying to deliver patient care in these new circumstances.

We got the consent and managed to see 5 of the 11 patients (45%). Those who were seen had satisfaction with the process, and were thankful to us.

We were able to diagnose two patients with likely Parkinson’s disease (as it was not possible to assess their rigidity, cogwheeling or postural instability hence the word likely is being used here).

One patient had already been diagnosed by someone, which was confirmed by our clinic. The remaining two were already diagnosed in previous consultations but needed a decision on initiation and optimisation of medications.

## STEPS TO ACHIEVE OPTIMUM AA CLINICS

### Preclinic (planning)

1. Patients were instructed to carry out basic observations, that is, their blood pressure. Fortunately, all of the patients had access to the blood pressure machine and they checked their blood pressure including for postural drop following telephonic instruction by our office. With time, we have developed written instruction, which is now sent to patients how to check for postural drop.
2. They were instructed to prepare a list of their questions to make the consultation focused and efficient as it could be overwhelming for some of the patients.
3. They were asked to have the medication list handy.
4. Mutually agreed time between the clinic hours was selected keeping in mind the convenience of family members too.
5. A technological dry run was done before every consultation; the camera, audio, speakers were tested beforehand.
6. A sign of ‘Do not Disturb’ was displayed on the door so that disruption was minimised.
7. Through video consultation, we were able to observe/do the following:
  - Facial appearance, speech, bradykinesia, transfer, assessing non-motor symptoms, gait including the 360° turns, arm swings, glabellar tap sign were observed with the help of family members (although not a very reliable sign) and 6-item cognitive impairment test (6-CIT) for memory issues.
  - Good discussion on activities of daily living—that allowed one to make a decision to initiate or alter the medications.
  - The consultation time was generally longer (roughly 50 min) than a traditional clinic.
  - In this consultation, likely diagnosis, management of the condition and medication side effects were discussed as one would discuss in the normal clinics.
  - Unfortunately, not able to assess rigidity, cogwheeling or postural instability as alluded earlier.
  - Routine blood tests including thyroid function test, haematinics, CT head or DaTscan (dopamine transporter single photon emission) were organised to get a better picture for those which were new suspected diagnoses.

## PROBLEMS WITH THE VIDEO CONSULTATION

There were few problems with this new service like initial echo on one of the consultations, which soon settled once family members were asked to mute their mobile phones; while on another occasion, a family member was not able to use the screen share application on the system, hence kept popping the head in the patient’s screen which led to distraction in the consultation. This needs better information on how to share the screen in future consultation. On another occasion, the whole system froze for a couple of minutes then returned later, a technical glitch. On another occasion, the video images started to deteriorate. This was secondary to bandwidth issues. Grandchildren simultaneously using video games was eating up the Wi-Fi bandwidth. It was easily resolved once grandchildren stopped the games.

## CONCLUSION

There was an assumption that the elderly population may not be able to adapt to video consultations, but this was not accurate. Those patients who were seen in the AA clinic had a positive feedback on this process.

Telephone consultation is quite acceptable and popular in the elderly population, but with time, video consultation would become the norm. Of course, help from family members is paramount.

Good planning beforehand is very advantageous for a desired outcome.

Reasonably good clinical examination was done through video consultation.

It is advantageous to have a blood pressure machine with the patients. Fortunately, each of the patient in our study had the machine and they managed not only to record the blood pressure but also checked for the postural drop on our instructions.

6-CIT was done easily.

Relevant investigations were organised to support the clinical suspicion.

Although this study is based on a small number of patients, but it seems video consultation will have a strong presence in the future. These are opportunities for innovation. No doubt the tech companies will soon develop user-friendly mobiles, tablets, iPads, smartphones or even desktop for elderly persons.

Unfortunately, it will have a knock-on effect on the employment for the outpatient staff. I can see that outpatient clinics will be truncated and trimmed with (may be) some job losses or redeployment. This will also see a drop in car parking and cafeteria revenues. But it will see release of space, which could be advantageous to the wider NHS.

Sooner or later, innovative ways of consultation will be part of teaching to medical students in the medical schools across the UK. Presently, some of the medical students in specialised branches are learning by attending the telephone clinics. It is better to be proactive rather reactive.

